# Supplementary material for: Fibrinogen induces neural stem cell differentiation into astrocytes in the subventricular zone via BMP signaling
Source: Nat Commun. 2020 Jan 31;11:630. doi: 10.1038/s41467-020-14466-y (PMC6994610; doi:10.1038/s41467-020-14466-y)
Supplement: Supplementary file 1 — Supplementary Information [file 41467_2020_14466_MOESM1_ESM.pdf]

Fibrinogen induces neural stem cell differentiation into astrocytes in the subventricular zone via BMP signalling

Pous *et al.*

Supplementary Information

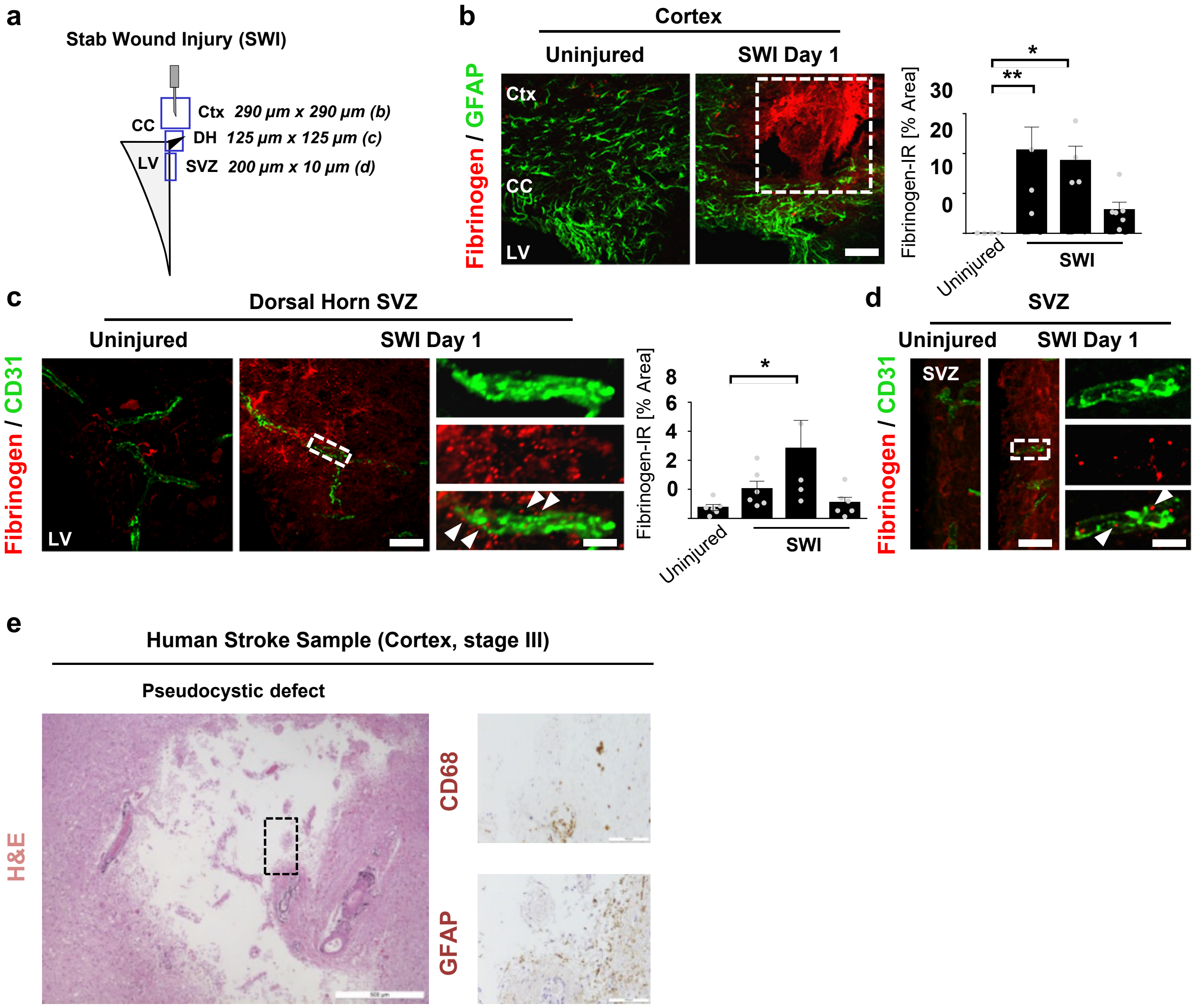

Supplementary Figure 1. Fibrinogen deposition in the SVZ after cortical stab wound injury. a Scheme illustrating the areas of analysis after stab wound injury. The blue boxes indicate the magnified areas analyzed in the figures b, c and d. **b** GFAP+ astrocytes (green) and fibrinogen (red) in the cortex 1 day after SWI and uninjured mice. The white box indicates the quantification area for fibrinogen immunoreactivity. Scale bar, 70 µm. Quantification of fibrinogen immunoreactivity in the cortex per area at different time points after SWI (n=4, mean±s.e.m, one-way ANOVA and Bonferroni's multiple comparisons test, *P<0.05, **P<0.01). **c** CD31+ blood vessels (green) and fibrinogen (red) in the dorsal horn of the SVZ injured mice 1 day after SWI and uninjured mice. The white box indicates the enlargement of a blood vessel with extravascular fibrinogen deposition (white arrows) 1 day after SWI (right). Scale bars, 21 µm, left, 7 µm, enlargement. Quantification of fibrinogen immunoreactivity in the dorsal horn of the SVZ per area at different time points after SWI (n=4, mean ±s.e.m, one-way ANOVA and Bonferroni's multiple comparisons test, *P<0.05). **d** Representative images of CD31+ blood vessels (green) and fibrinogen (red) in the SVZ 1 day after SWI and uninjured mice. The white box indicates the enlargement of a blood vessel with extravascular fibrinogen deposition (white arrows) 1 day after SWI (right) (n=4). Scale bar, 28 µm, left, 8 µm, enlargement. **e** Hematoxylin/Eosin (H&E) labeling of the cortical stroke region of a patient (stadium III) (left). Black box indicating enlargement of CD68+ macrophages (right top) and GFAP+ astrocytes (right bottom) surrounding the stroke area. SVZ, subventricular zone, LV, lateral ventricle, Ctc, cortex, CC, corpus callosum.

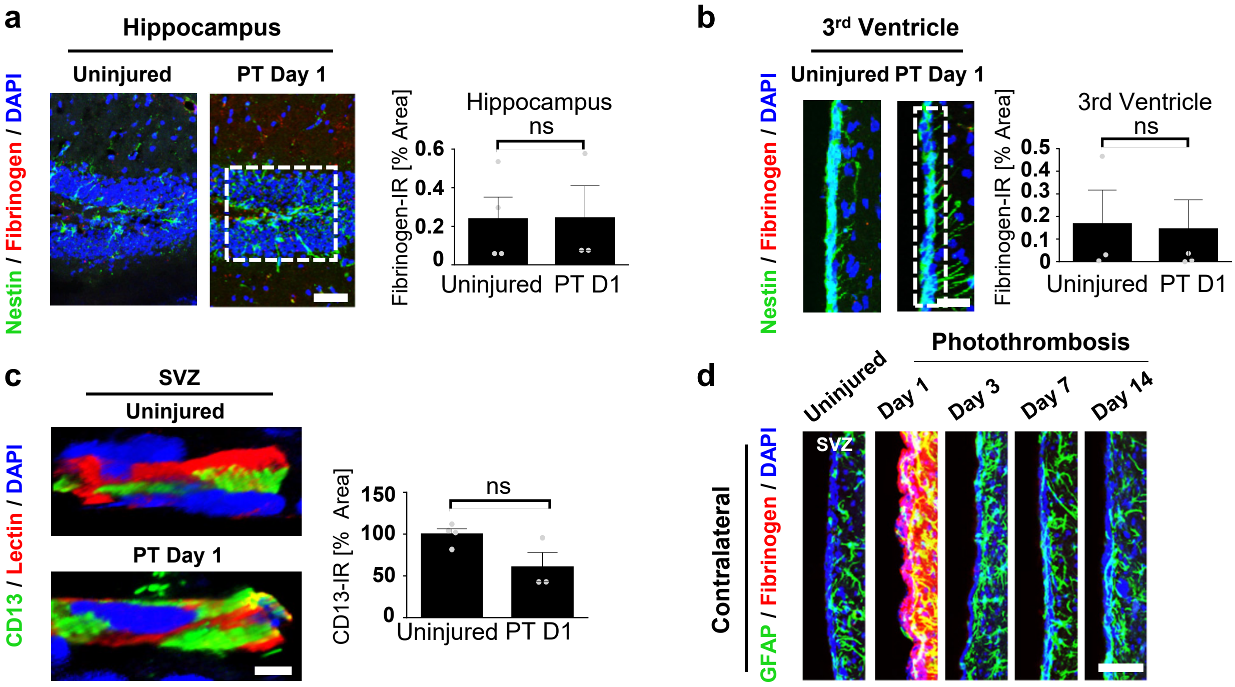

Supplementary Figure 2. Fibrinogen deposition after cortical injury is specific to the SVZ stem cell niche. a Nestin+ NSPCs (green) and fibrinogen (red) in the dentate gyrus of the hippocampus of uninjured and injured mice 1 day after photothrombotic ischemia. The white box represents the quantification area for fibrinogen immunoreactivity. Scale bar, 51 µm, left (*n*=4 mice, mean±s.e.m, unpaired Student's *t* test, ns, not significant). **b** Nestin+ NSPCs (green) and fibrinogen (red) lining the third ventricle 1 day after photothrombotic ischemia. The white box represents the quantification area for fibrinogen immunoreactivity. Scale bar, 22 µm. Quantification for fibrinogen immunoreactivity around the third ventricle per area (*n*=3 for uninjured, n=4 for PT Day 1 mice, mean±s.e.m, unpaired Student's *t* test, ns, not significant). **c** CD13+ pericytes (green) and lectin+ blood vessels (red) in the SVZ of WT uninjured and injured mice 1 day after PT. Scale bar, 4 µm. Quantification for CD13 immunoreactivity (i.e. pericyte coverage) around the blood vessels in the SVZ of uninjured and injured mice (PT Day 1) (*n*=3 mice, mean±s.e.m., unpaired Student's *t* test, ns, not significant). **d** Representative images of GFAP+ astrocytes (green) and fibrinogen (red) in the contralateral SVZ at different time points after photothrombotic ischemia (*n*=4 mice). Scale bar, 57 µm. SVZ, subventricular zone.

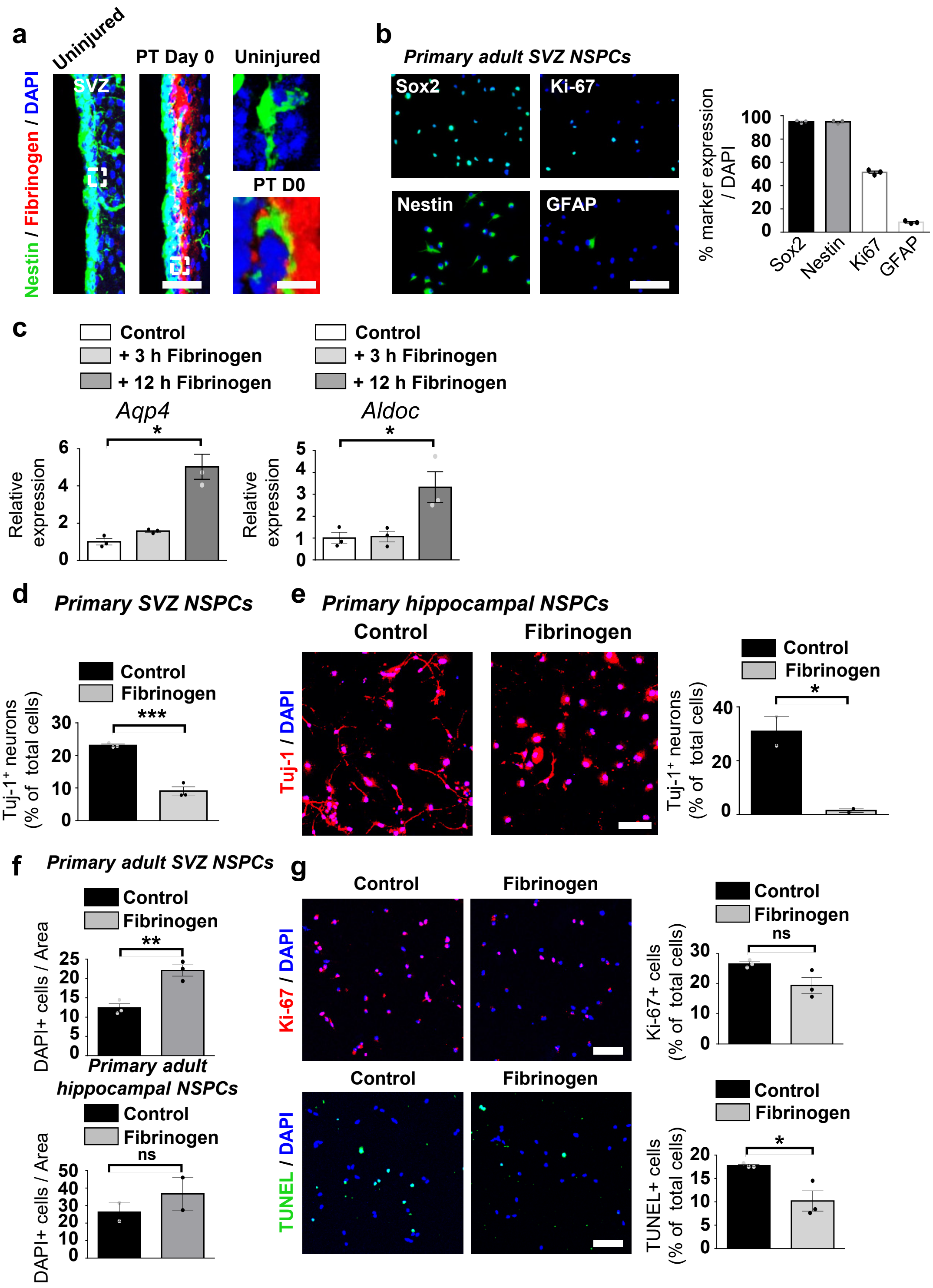
a
Uninjured
PT Day 0
Uninjured
PT D0
SVZ
Nestin / Fibrinogen / DAPI
b
Primary adult SVZ NSPCs
Sox2
Ki-67
Nestin
GFAP
% marker expression / DAPI
100
80
60
40
20
0
Sox2
Nestin
Ki67
GFAP
c
Control
+ 3 h Fibrinogen
+ 12 h Fibrinogen
Aqp4
Aldoc
Relative expression
*
d
Primary SVZ NSPCs
Control
Fibrinogen
***
Tuj-1+ neurons (% of total cells)
e
Primary hippocampal NSPCs
Control
Fibrinogen
Tuj-1 / DAPI
*
f
Primary adult SVZ NSPCs
DAPI+ cells / Area
**
Primary adult hippocampal NSPCs
ns
g
Ki-67 / DAPI
Ki-67+ cells (% of total cells)
TUNEL / DAPI
TUNEL+ cells (% of total cells)

Supplementary Figure 3. Promotion of fibrinogen-induced NSPCs towards astrogenesis over neurogenesis. **a** Representative images of nestin+ NSPCs (green) and fibrinogen (red) in the SVZ stem cell niche 5 h after photothrombotic ischemia (PT Day 0). The white boxes indicate the enlargement of fibrinogen deposition in the stem cell niche environment (right, bottom) compared to no fibrinogen deposition in the uninjured control (right, top). Scale bars, 36 µm, left and 5 µm, enlargement. **b** Sox2, Ki-67, Nestin and GFAP (green) immunostainings in untreated adult SVZ-derived primary NSPC cultures 3 hours after initiation of differentiation. Scale bar: 100 µm. Quantification of the number of cells positive for the individual NSPC marker. Results are from 3 independent experiments (mean±s.e.m). **c** *Aqp4* (left) and *Aldoc* (right) mRNA expression in NSPCs after 3 h and 12 h of fibrinogen treatment determined by quantitative PCR and normalized to *GAPDH* (n=3 mice, mean±s.e.m, unpaired Student's t test, *P<0.05). **d** Quantification of Tuj-1+ neurons in untreated and fibrinogen-treated adult SVZ-derived WT NSPC cultures after 4 days on poly-D-lysine. (n=3 mice, mean±s.e.m., unpaired Student's t test, ***P<0.001). **e** Tuj-1+ neurons (red) in untreated and fibrinogen-treated adult hippocampal NSPC cultures after 7 days on poly-D-lysine/laminin. Scale bar, 104 µm. Quantification of Tuj-1+ neurons of fibrinogen-treated adult hippocampal NSPCs compared to untreated NSPCs. Results are from 2 independent experiments performed in duplicate (mean±s.e.m., unpaired Student's t test, *P<0.05). **f** Quantification of the DAPI+ nuclei in fibrinogen-treated SVZ-derived primary NSPCs (top) and hippocampal-derived NSPCs (bottom) compared to untreated NSPCs after 4 days of differentiation. Results are from 3 independent experiments (top) and 2 independent experiments performed in duplicates (bottom). (mean±s.e.m., unpaired Student's t test, **P<0.01, ns not significant). **g** Ki-67+ proliferating cells (red, top) and TUNEL+ apoptotic cells (green, bottom) of untreated and fibrinogen-treated adult SVZ-derived NSPCs cultured for 1 day on poly-D-lysine. Scale bars, 104 µm. Quantification of Ki-67+ proliferating (top) and TUNEL+ apoptotic (bottom) NSPCs treated with fibrinogen compared to untreated cells after 1 day on poly-D-lysine. Results are from 3 independent experiments (mean±s.e.m., unpaired Student's t test, P<0.05, ns=not significant). SVZ, subventricular zone.

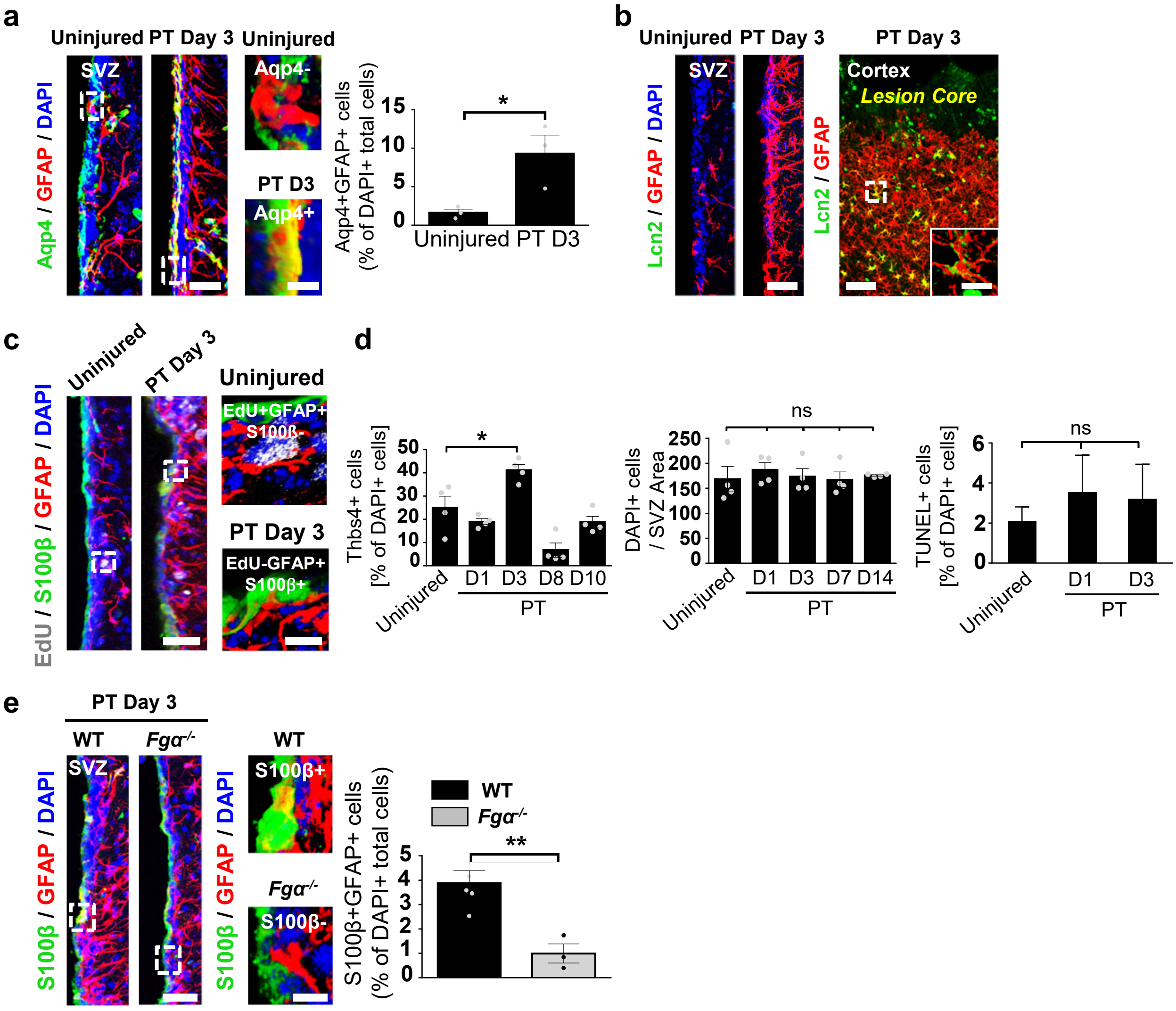

Supplementary Figure 4. Fibrinogen depletion reduces SVZ astrogliogenesis after PT. a Aqp4 (green) and GFAP (red) immunostainings in the SVZ of uninjured and injured mice 3 days after PT. The white boxes indicate the enlargement of Aqp4-GFAP+ (right, top) and Aqp4+GFAP+ (right, bottom) cells in the SVZ of uninjured and injured mice, respectively. Scale bar, 26 µm, left, 5 µm, enlargement. Quantification of the Aqp4+GFAP+ astrocytes in the SVZ after PT (*n*=3 mice, mean±s.e.m., unpaired Student's *t* test, **P*<0.05). **b** Representative images of Lcn2 (green) and GFAP (red) immunostainings in the SVZ (left) of uninjured and injured mice and in the cortex of injured mice (right) 3 days after PT. The white box indicates the enlargement of a Lcn2+GFAP+ cell in the cortex of injured mice (*n*=3 mice). Scale bars, 22 µm, left, 116 µm, right, 10 µm, enlargement. **c** Representative images of EdU (grey), S100β (green) and GFAP (red) immunostainings in the SVZ at 3 days after PT. The white boxes indicate the enlargement of an EdU+GFAP+S100β- (right, top) and EdU-S100β+GFAP+ (right, bottom) cell in the SVZ of uninjured and injured mice (PT day 3), respectively (n=4 mice). Scale bar, 25 µm. 5 µm, enlargement. **d** Quantifications of Thbs4+ cells (left) and DAPI+ cells (middle) and TUNEL+ cells (right) in the SVZ at different time points after PT (n=3 (left), n=4 (middle), n=4 mice (right), mean±s.e.m., one-way ANOVA and Bonferroni's multiple comparisons test, *P<0.05, ns, not significant). **e** S100β (red) and GFAP (green) immunostainings in the SVZ of *Fgα*$^{-/-}$ mice compared to WT mice 3 days after photothrombotic ischemia. The white boxes indicate the enlargement of a S100β+GFAP+ and a S100β-GFAP+ cell in the SVZ of WT (top) and *Fgα*$^{-/-}$ (bottom) mice, respectively, 3 days after PT. Scale bars, 36 µm, left and 7 µm, enlargment. Quantification of S100β+GFAP+ cells in the SVZ per area (*n*=5 WT mice, *n*=3 injured *Fgα*$^{-/-}$ mice, mean±s.e.m., unpaired Student's *t* test ***P*<0.01). SVZ, subventricular zone.

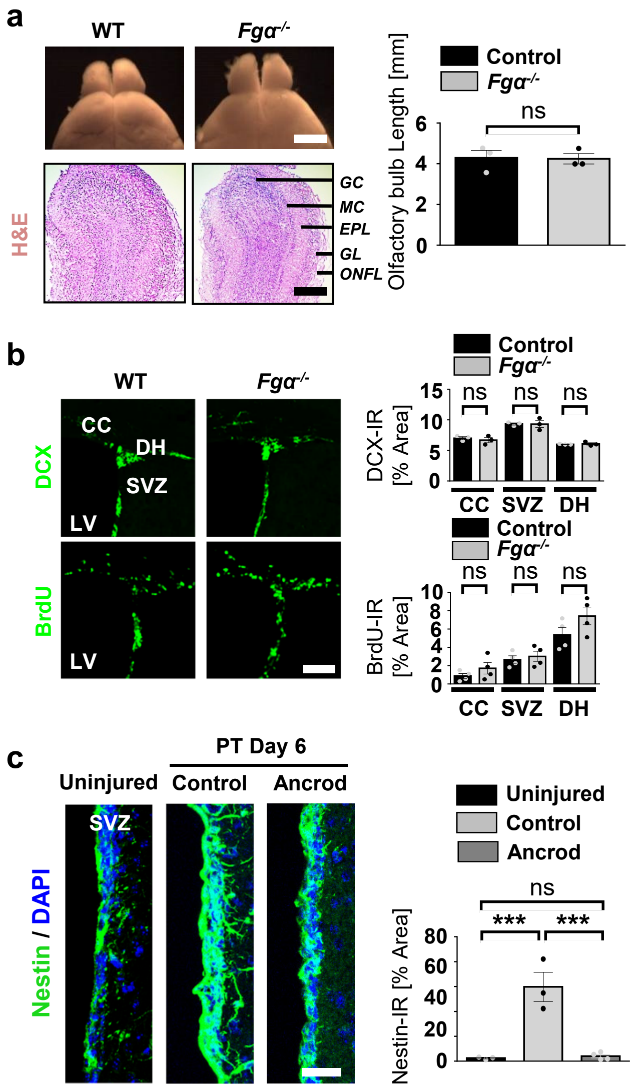

Supplementary Figure 5. Unaffected SVZ NSPC pool after genetic and pharmacologic depletions of fibrinogen. **a** Overview of olfactory bulb images of WT and *Fgα*$^{-/-}$ mice (top) and Hematoxylin/Eosin (H&E) labeling of WT and *Fgα*$^{-/-}$ mice (bottom). Scale bars, 5.7 mm, top, 329 µm, bottom. Quantification of olfactory bulb size of *Fgα*$^{-/-}$ and WT control mice. (*n*=3 mice, mean±s.e.m., unpaired Student's *t* test, ns, not significant). **b** DCX+ (top) and BrdU+ (bottom) NSPCs labeling of uninjured *Fgα*$^{-/-}$ and WT control mice. Scale bar, 260 µm. Quantification of DCX and BrdU immunoreactivity in the corpus callosum (CC), the dorsal horn of the SVZ (DH-SVZ) and the SVZ of uninjured *Fgα*$^{-/-}$ and WT control mice. (*n*=4 mice, mean±s.e.m,, one-way ANOVA and Bonferroni's multiple comparisons test, ns, not significant). **c** Nestin (green) immunostaining in the SVZ of ancrod-treated and control mice 6 days after PT compared to uninjured WT mice. Scale bar, 33 µm. Quantification of nestin immunoreactivity of ancrod-treated and control mice 6 days after PT compared to uninjured WT mice (control PT *n=3,* ancrod PT *n=4,* uninjured *n=3* mice, mean±s.e.m., one-way ANOVA and Bonferroni's multiple comparisons test ***P<0.001, ns, not significant). LV, Lateral Ventricle; CC, Corpus Callosum; CTX, Cortex; DH, Dorsal Horn, SVZ, subventricular zone.

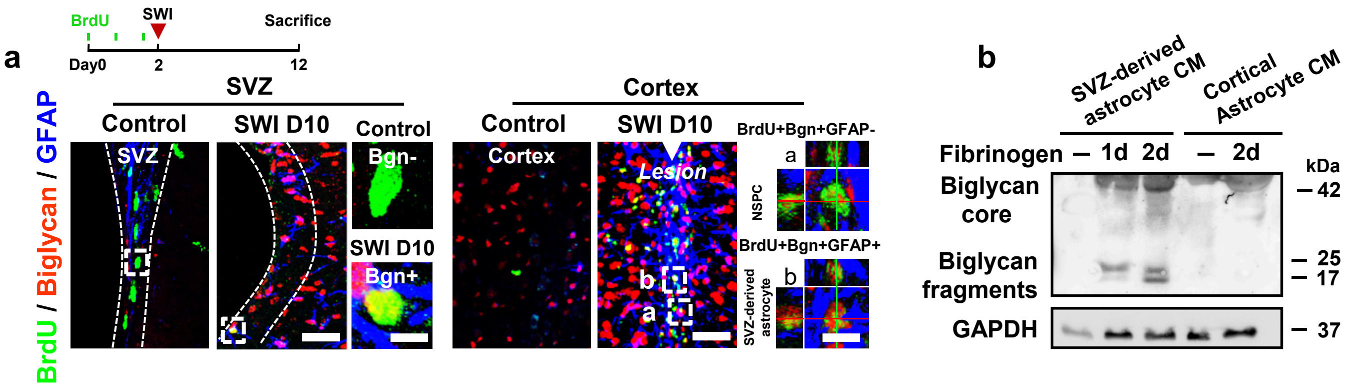

Supplementary Figure 6. Fibrinogen induces NSPC expression and secretion of biglycan. a Immunolabeling for biglycan (red), GFAP (blue) and BrdU (green) in the SVZ of WT mice 10 days after SWI compared to control mice (left). White boxes indicate the enlargement of biglycan-GFAP-BrdU+ and biglycan+GFAP+BrdU+ cells in the SVZ of control mice (right, top) and mice 10 days after SWI (right, bottom), respectively. Scale bars, 30 µm, left and 8 µm, enlargement. Representative images of n=3 mice. Immunolabeling for biglycan (red), GFAP (blue) and BrdU (green) in the cortex of WT mice 10 days after SWI compared to control mice (right). White boxes indicate the enlargement of biglycan+GFAP-BrdU+ (a, right, top) and biglycan+GFAP+BrdU+ (b, right, bottom) SVZ-derived NSPCs and SVZ-derived newborn astrocytes in the lesion area of mice 10 days after SWI, respectively. Scale bars, 30 µm, left and 7 µm, enlargement. Representative images of n=3 mice. **b** Western blot analysis of biglycan protein expression in supernatant of NSPCs or cortical astrocytes after fibrinogen treatment. Representative immunoblots from three independent experiments are shown. SVZ, subventricular zone.

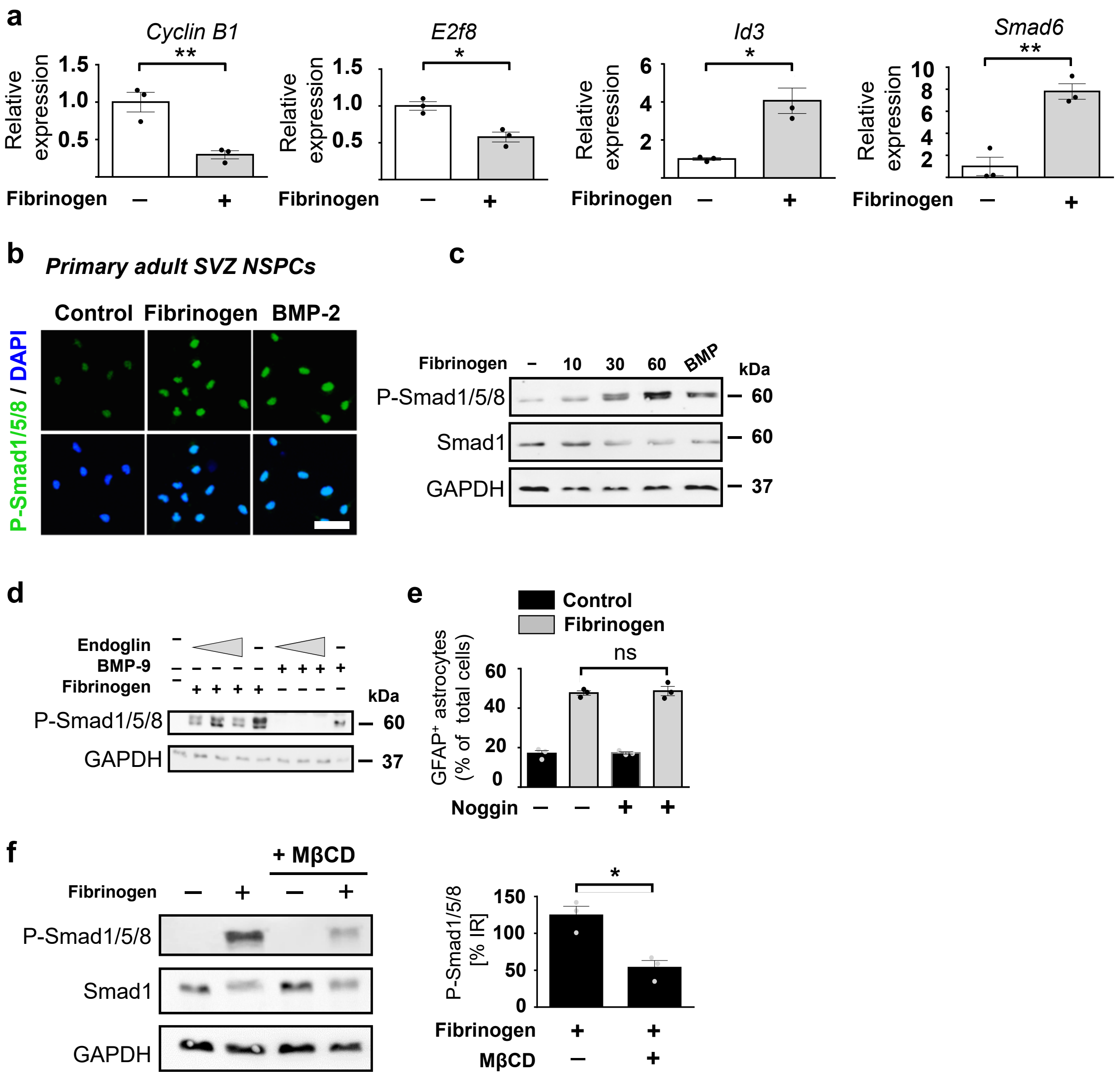

Supplementary Figure 7. Fibrinogen activates BMP type I receptor signaling via lipid rafts. a Expression of *Cyclin B1, E2f8, Id3 and Smad6* mRNAs in NSPCs after 12 h of fibrinogen treatment determined by quantitative PCR and normalized to *GAPDH*. Results are from 3 independent experiments (mean±s.e.m., unpaired Student's *t* test, **P*<0.05, ***P*<0.01). **b** P-Smad1/5/8 immunolabeling (green) of NSPCs treated for 1 hour with fibrinogen compared to untreated cells. BMP-2 treatment served as positive control. Scale bar, 58 µm. **c** Immunoblot for P-Smad1/5/8 and Smad1 in NSPCs treated with fibrinogen for 10, 30 or 60 minutes or left untreated. BMP-2 served as positive control. Representative immunoblot from 3 independent experiments is shown. **d** Immunoblot for P-Smad1/5/8 in NSPCs pretreated with increasing concentration of endoglin inhibitor 1 h before fibrinogen treatment. BMP-9 served as positive control. A representative immunoblot from 2 independent experiments is shown. **e** Quantification of GFAP+ astrocytes derived from differentiated WT NSPCs pretreated with Noggin 1 h before fibrinogen treatment. Results from 3 independent experiments are shown (mean±s.e.m., one-way ANOVA and Bonferroni's multiple comparisons test, ns, not significant). **f** Immunoblot for P-Smad1/5/8 and Smad1 in NSPCs pretreated with the lipid raft inhibitor MβCD 5 h before fibrinogen treatment. Quantification of P-Smad1/5/8 immunoreactivity normalized to GAPDH loading control. Results are from 3 independent experiments (mean±s.e.m., unpaired Student's *t* test, **P*<0.05).

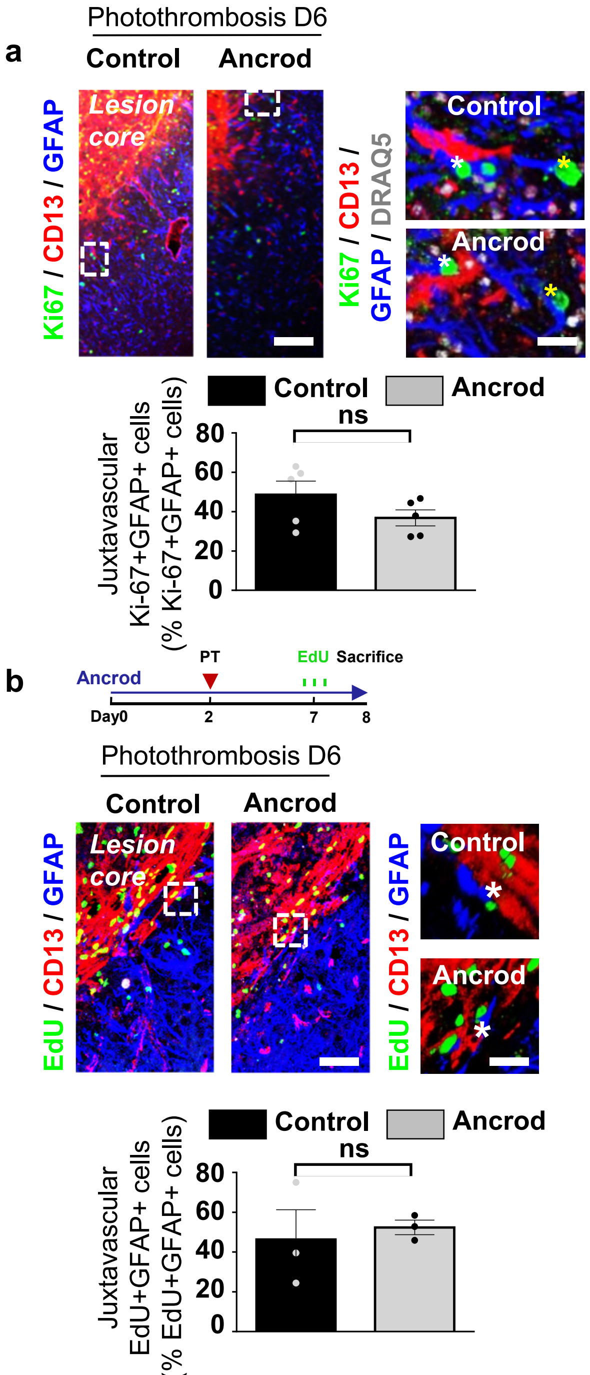

Supplementary Figure 8. Fibrinogen does not affect juxtavascular astrocyte proliferation after PT. a Ki-67 (green), CD13 (red), GFAP (blue) immunostainings in the lesion area of fibrinogen-depleted mice (Ancrod) compared to controls (NaCl) at 6 days after PT. The white boxes indicate the enlargement of a Ki67+GFAP+DRAQ5+ cell in direct contact with CD13+ blood vessel in ancrod and control mice (right). The white and yellow stars point out juxtavascular and non juxtavascular proliferating astrocytes, respectively. Quantification of juxtavascular Ki67+GFAP+ astrocytes in ancrod and control mice (n=5 mice, mean±s.e.m., unpaired Student's *t* test, ns, not significant). **b** EdU (green), CD13 (red), GFAP (blue) immunostainings in the lesion area of fibrinogen-depleted mice (Ancrod) compared to controls (NaCl) at 6 days after PT. The white boxes indicate the enlargement of a EdU+GFAP+ cell in direct contact with CD13+ blood vessel in ancrod and control mice (right). The white stars point out juxtavascular newborn astrocytes, respectively. Quantification of juxtavascular EdU+GFAP+ astrocytes in ancrod and control mice (n=4 mice, mean±s.e.m., unpaired Student's *t* test, ns, not significant).

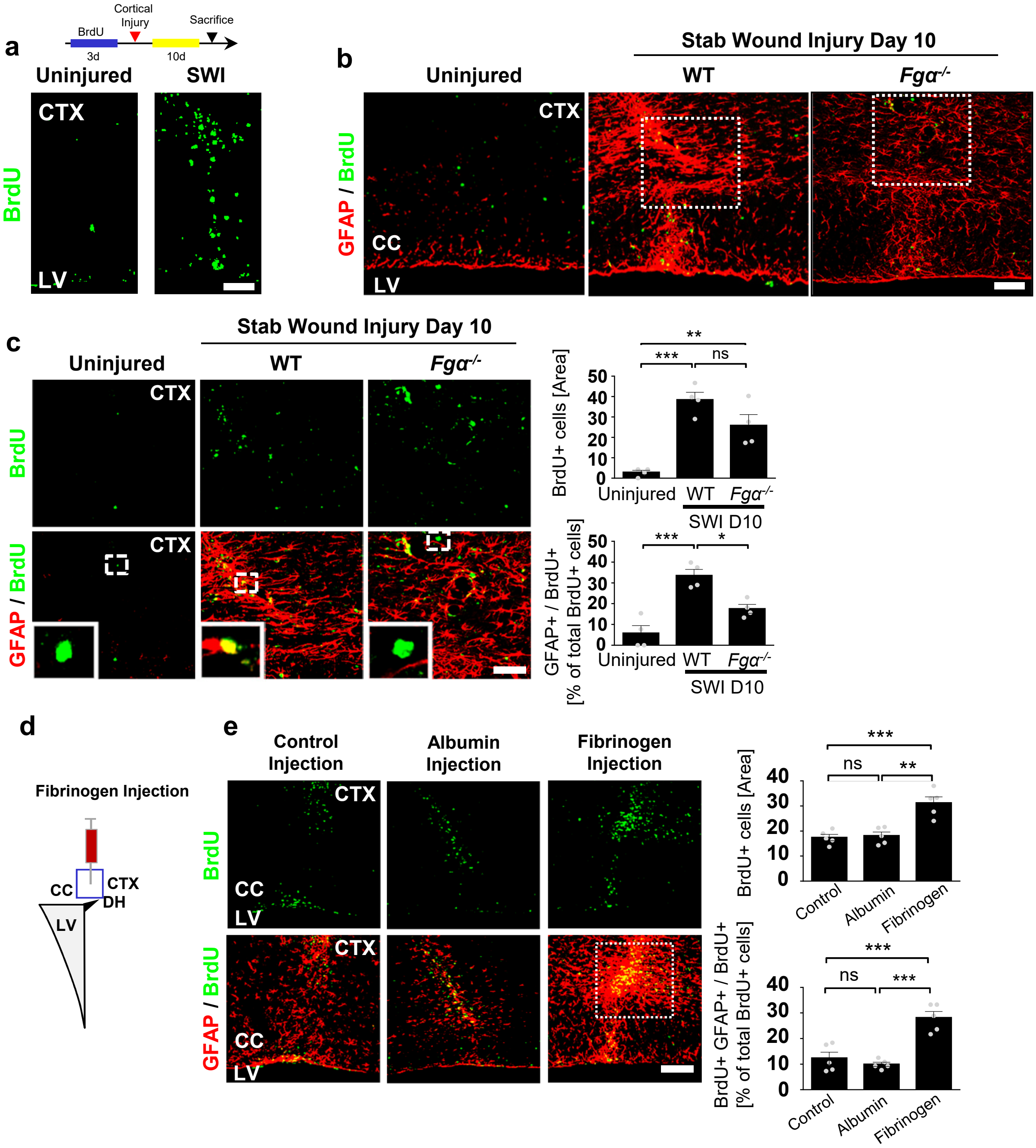

Supplementary Figure 9. Fibrinogen drives the SVZ-derived astrocyte contribution to the lesion scar. a Experimetal setup for stab wound injury on BrdU-injected *WT* mice (top). Representative images of BrdU (green) immunostaining in the cortex of mice 10 days after SWI compared to uninjured mice. Scale bar: 55 µm. **b** Representative images of BrdU (green) and GFAP (red) immunostainings in uninjured WT mice and injured WT and $Fg\alpha^{-/-}$ mice in the cortex 10 days after SWI. The white boxes indicate the quantified areas shown in **c**. Scale bar: 80 µm. **c** BrdU (green) and GFAP (red) immunostainings in uninjured WT mice and injured WT and $Fg\alpha^{-/-}$ mice in the lesion area 10 days after SWI. The white boxes indicate the enlargement of BrdU+GFAP- cells in uninjured WT and injured $Fg\alpha^{-/-}$ mice and a BrdU+GFAP+ cell in a injured WT mouse. Scale bar: 40 µm. Quantification of BrdU$^+$ (top) and BrdU$^+$GFAP$^+$ (bottom) cells in WT and $Fg\alpha^{-/-}$ mice at 10 days after SWI compared to uninjured WT mice (n=4 mice, mean±s.e.m., one-way ANOVA and Bonferroni's multiple comparisons test, ***P< 0.001, **P < 0.01, *P < 0.05 and ns, not significant). **d** Experimental setup for stereotactic injection of fibrinogen into the cortex. **e** BrdU (green) and GFAP (red) immunostainings of WT mice injected with NaCl (Control), albumin or fibrinogen in the cortex. The white box indicates the quantification area. Scale bar, 140 µm. Quantification of BrdU+ (top) and BrdU+GFAP+ (bottom) cells (n=4 mice, mean±s.e.m., one-way ANOVA and Bonferroni's multiple comparisons test, ***P<0.001, **P<0.01 and ns, not significant). LV, Lateral Ventricle; CC, Corpus Callosum; CTX, Cortex; DH, Dorsal Horn.

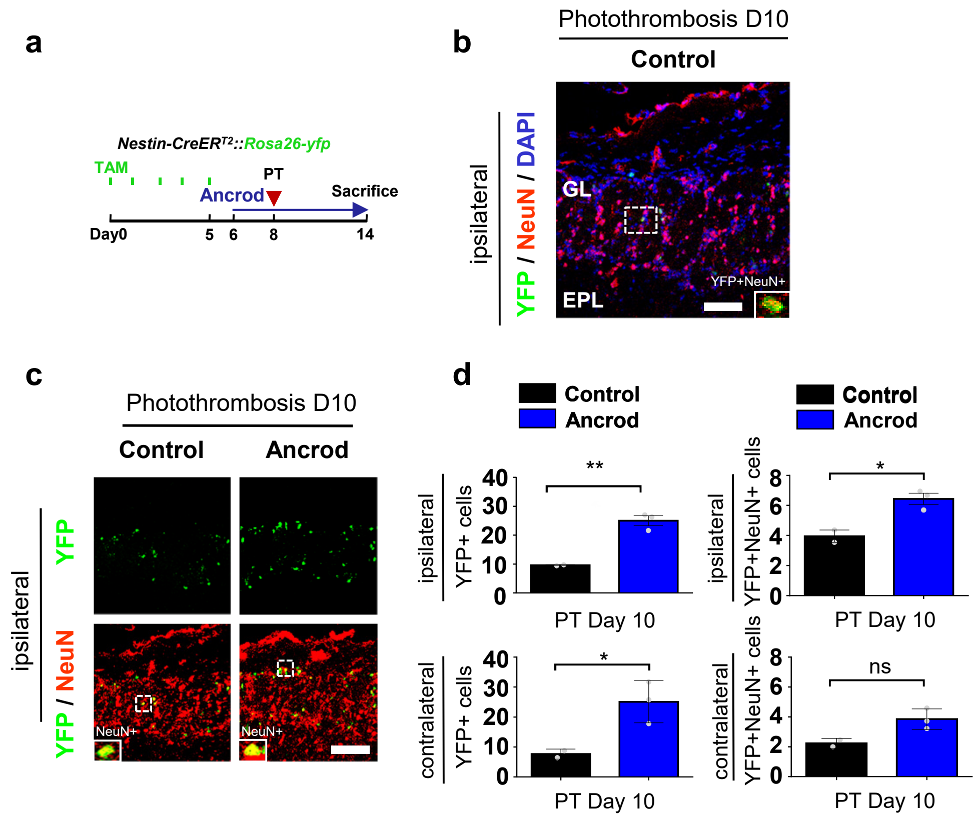

Supplementary Figure 10. Fibrinogen depletion increases the SVZ-derived neuron contribution in OBs after PT. a Experimetal setup for ancrod-administered *Nestin-CreER^{T2}::Rosa26-yfp* mice. TAM: tamoxifen (top). **b** Representative image of the OB of control (NaCl) treated WT mice with immunolabeling for YFP (green) and NeuN (red) 10 days after PT. **c** High magnification images of the glomerular layer of the OB of ancrod treated and control WT mice with immunolabeling for YFP (green) and NeuN (red) 10 days after PT. **d** Quantification of YFP+ cells and of YFP+NeuN+ cells of ancrod and control mice (n=2 control mice, n=3 ancrod mice, unpaired Student's *t*-test, *P<0.05, **P<0.01, ns, not significant). EPL, external plexiform layer, GL, glomerular layer.

Pous et al., Supplementary Table 1

List of top differentially expressed genes in WT NSPCs 12 h after fibrinogen treatment

List of the top 215 upregulated genes

			WT 12h fibrinogen vs. WT untreated		
**Rank**	**ID-Ref**	**Gene Symbol**	**Fold-Change**	**P-value**	**Q-value**
1	10391798	Gfap	8,0	9,09E-14	2,59E-09
2	10518494	Ptchd2	7,3	4,62E-09	5,12E-07
3	10533050	Hspb8	6,3	1,20E-10	4,03E-08
4	10510299	Fbxo2	6,1	2,06E-11	1,68E-08
5	10581388	Lcat	6,0	4,76E-11	2,24E-08
6	10462507	Papss2	5,9	1,96E-09	2,74E-07
7	10477169	Id1	5,8	1,40E-11	1,44E-08
8	10456392	Cidea	5,5	1,64E-07	8,01E-06
9	10471154	Gm5424 : Ass1	4,9	1,10E-10	3,83E-08
10	10454856	Psd2	4,6	2,11E-10	5,67E-08
11	10600169	Bgn	4,5	3,51E-08	2,36E-06
12	10351430	Rxrg	4,4	4,86E-06	1,08E-04
13	10457614	Aqp4	4,4	2,38E-11	1,69E-08
14	10492091	Smad9	4,3	5,22E-11	2,36E-08
15	10369932	Susd2	4,3	6,46E-06	1,34E-04
16	10543428	Iqub	4,2	6,41E-10	1,28E-07
17	10509838	Padi2	4,2	5,31E-11	2,36E-08
18	10469066	Ccdc3	4,0	1,87E-08	1,48E-06
19	10512919	Grin3a	3,9	4,55E-07	1,71E-05
20	10391828	C1ql1	3,9	7,12E-10	1,39E-07
21	10530059	Sel1l3	3,8	3,70E-10	8,49E-08
22	10464030	Adra2a	3,7	4,64E-09	5,12E-07
23	10392464	Fam20a	3,7	1,13E-09	1,87E-07
24	10545372	Atoh8	3,4	5,61E-10	1,16E-07
25	10429802	Plec	3,4	1,78E-09	2,54E-07
26	10517287	Man1c1	3,4	7,36E-07	2,52E-05
27	10535065	Adap1	3,3	1,20E-05	2,16E-04
28	10383717	Inpp5j	3,3	2,60E-06	6,64E-05
29	10521415	Ablim2	3,2	1,70E-10	4,99E-08
30	10399465	Fam84a	3,2	9,88E-09	9,25E-07
31	10404836	Rnf182	3,1	3,65E-08	2,43E-06
32	10480035	Pfkfb3	3,1	1,42E-08	1,21E-06
33	10470959	Phyhd1	3,1	4,70E-09	5,15E-07
34	10454851	Cxxc5	3,1	2,56E-07	1,10E-05
35	10437205	Pcp4	3,0	1,02E-05	1,92E-04
36	10539111	Tmem150a	3,0	3,09E-08	2,17E-06
37	10494643	Hmgcs2	3,0	2,20E-09	2,98E-07
38	10495035	Slc16a1	3,0	5,83E-06	1,24E-04
39	10434089	Ccdc74a	3,0	5,81E-06	1,24E-04
40	10371379	Nuak1	2,9	6,98E-07	2,43E-05
41	10472923	Ak4	2,9	7,82E-09	7,76E-07
42	10502655	Cyr61	2,9	7,99E-08	4,58E-06

43	10470214	Kcnt1	2,9	2,43E-08	1,80E-06
44	10364784	Reep6	2,8	7,14E-07	2,47E-05
45	10523021	Slc4a4	2,8	4,11E-09	4,73E-07
46	10459530	B430212C06Rik	2,8	1,05E-06	3,30E-05
47	10455967	2610318N02Rik	2,8	4,91E-09	5,35E-07
48	10351465	1700084C01Rik	2,8	1,60E-08	1,33E-06
49	10421730	AU021034	2,8	2,44E-07	1,05E-05
50	10569020	Ifitm6	2,8	1,05E-08	9,62E-07
51	10451225	Polh	2,8	4,18E-08	2,73E-06
52	10416057	Clu	2,8	2,12E-09	2,88E-07
53	10607738	Car5b	2,8	9,11E-09	8,72E-07
54	10574166	Cpne2	2,7	9,18E-07	2,97E-05
55	10408850	Nedd9	2,7	2,89E-07	1,21E-05
56	10394685	Ntsr2	2,7	2,44E-07	1,06E-05
57	10601729	Drp2	2,7	1,93E-06	5,28E-05
58	10587818	Plscr4	2,7	1,10E-09	1,84E-07
59	10600210	Slc6a8	2,7	1,27E-08	1,11E-06
60	10373768	Selm	2,7	4,27E-04	0,002911993
61	10571444	Slc7a2	2,6	8,77E-06	1,71E-04
62	10587799	Plscr2	2,6	6,85E-09	7,11E-07
63	10474229	Cd59a : Cd59b	2,6	2,80E-05	3,99E-04
64	10392415	Rgs9	2,6	1,19E-06	3,64E-05
65	10460400	Pcx	2,6	8,90E-09	8,67E-07
66	10397962	Fam181a	2,6	5,91E-07	2,11E-05
67	10580473	Zfp423	2,6	1,12E-07	5,95E-06
68	10404975	Id4	2,5	2,26E-09	3,05E-07
69	10345411	Arhgef4	2,5	9,46E-08	5,26E-06
70	10372730	Iltifb	2,5	2,04E-06	5,53E-05
71	10514300	Ifna12	2,5	1,01E-04	0,001009845
72	10453233	Slc8a1	2,5	2,91E-07	1,21E-05
73	10469389	Slc39a12	2,5	6,27E-08	3,76E-06
74	10458560	Fgf1	2,5	1,08E-07	5,80E-06
75	10530421	Gabra4	2,5	1,30E-04	0,001212414
76	10595680	Tbc1d2b	2,5	1,72E-07	8,26E-06
77	10440903	4932438H23Rik	2,4	4,04E-09	4,69E-07
78	10409261	Diras2	2,4	1,15E-04	0,001118226
79	10565627	Aqp11	2,4	5,91E-05	6,81E-04
80	10510129	Dhrs3	2,4	1,65E-06	4,66E-05
81	10528385	Reln	2,4	6,48E-07	2,28E-05
82	10502863	Ak5	2,4	2,56E-06	6,59E-05
83	10381298	Ramp2	2,4	2,96E-06	7,32E-05
84	10389816	Tom1l1 : Cox11	2,4	7,83E-09	7,76E-07
85	10594044	Islr	2,4	3,32E-08	2,28E-06
86	10509163	Id3	2,4	3,48E-09	4,21E-07
87	10548051	Kcna6	2,4	2,55E-09	3,34E-07
88	10562211	Fxyd1	2,4	1,88E-07	8,85E-06
89	10372528	Kcnmb4 : 1700058(	2,4	3,04E-06	7,46E-05
90	10417628	Cadps	2,4	1,19E-07	6,25E-06
91	10439542	Zdhhc23	2,4	1,34E-08	1,16E-06
92	10529875	Ldb2	2,3	1,95E-05	3,08E-04

93	10430818	Tnfrsf13c	2,3	7,27E-05	7,91E-04
94	10530819	Hopx	2,3	1,26E-08	1,11E-06
95	10429754	Nrbp2	2,3	6,18E-11	2,55E-08
96	10571865	Scrg1	2,3	3,34E-11	1,90E-08
97	10604175	Fam70a	2,3	4,71E-09	5,15E-07
98	10492330	P2ry1	2,3	4,07E-08	2,67E-06
99	10354003	Mgat4a	2,3	5,60E-05	6,54E-04
100	10406777	Gcnt4	2,3	7,36E-05	7,98E-04
101	10453390	Six3os1	1,96E-05	3,09E-04	2,3
102	10425623	Csdc2	1,04E-09	1,76E-07	2,3
103	10355806	Tuba4a	4,83E-08	3,08E-06	2,3
104	10488762	Snta1	9,62E-06	1,83E-04	2,3
105	10518735	Spsb1	1,10E-05	2,01E-04	2,3
106	10546829	Oxtr	6,42E-05	7,24E-04	2,3
107	10417972	Camk2g	1,99E-08	1,56E-06	2,3
108	10462603	Fas	4,87E-08	3,09E-06	2,3
109	10500204	Ecm1	9,13E-07	2,96E-05	2,2
110	10407173	Il6st	1,96E-08	1,53E-06	2,2
111	10357878	Adora1	3,58E-08	2,40E-06	2,2
112	10528702	Prkag2	1,17E-07	6,18E-06	2,2
113	10440344	Robo2	7,23E-07	2,49E-05	2,2
114	10400405	Nfkbia	3,56E-07	1,41E-05	2,2
115	10473432	Tnks1bp1	1,69E-07	8,19E-06	2,2
116	10398075	Serpina3n	9,09E-06	1,75E-04	2,2
117	10532538	Asphd2	1,40E-07	7,10E-06	2,2
118	10480275	Nebl	5,42E-09	5,84E-07	2,2
119	10444895	Flot1	3,37E-07	1,35E-05	2,2
120	10372648	Lyz2	3,45E-06	8,31E-05	2,2
121	10452151	Rfx2	4,12E-06	9,49E-05	2,2
122	10541354	A2m	8,87E-08	5,02E-06	2,2
123	10544062	D630045J12Rik	3,07E-06	7,51E-05	2,2
124	10360666	6330403A02Rik	1,43E-07	7,18E-06	2,2
125	10447697	6530411M01Rik	1,09E-06	3,40E-05	2,2
126	10594418	Smad6	6,28E-07	2,22E-05	2,2
127	10364502	Palm	1,47E-08	1,24E-06	2,2
128	10489484	Sdc4	1,19E-09	1,93E-07	2,2
129	10376241	Zic5	9,91E-07	3,16E-05	2,2
130	10357339	Gpr39 : Lypd1	4,29E-06	9,79E-05	2,2
131	10495675	F3	1,51E-07	7,50E-06	2,2
132	10553092	Sphk2 : Dbp	4,05E-07	1,57E-05	2,2
133	10583021	Pdgfd	1,66E-04	0,001453659	2,2
134	10433172	Glycam1	1,27E-06	3,82E-05	2,2
135	10430319	Tst	4,87E-08	3,09E-06	2,1
136	10352234	Itpkb	1,02E-09	1,73E-07	2,1
137	10575693	Vat1l	2,79E-05	3,98E-04	2,1
138	10546725	Pdzrn3	2,02E-07	9,29E-06	2,1
139	10546421	Prickle2	3,48E-07	1,39E-05	2,1
140	10431711	Slc2a13	1,05E-04	0,001039063	2,1
141	10476108	Ebf4	9,26E-09	8,81E-07	2,1
142	10500283	Car14 : Aph1a	3,89E-04	0,002717008	2,1

143	10466712	Mamdc2	1,93E-05	3,05E-04	2,1
144	10498576	Lxn	1,13E-08	1,02E-06	2,1
145	10345074	Cetn4	1,57E-06	4,49E-05	2,1
146	10418927	Bmpr1a	7,71E-07	2,62E-05	2,1
147	10440099	Dcbld2 : St3gal6	2,75E-07	1,16E-05	2,1
148	10595718	Chst2	1,46E-06	4,25E-05	2,1
149	10458340	Hbegf	3,76E-07	1,48E-05	2,1
150	10465314	Capn1	1,13E-04	0,00110047	2,1
151	10539238	Fam176a	2,33E-05	3,50E-04	2,1
152	10351131	Myoc	2,50E-06	6,49E-05	2,1
153	10540059	Slc41a3	4,58E-10	9,95E-08	2,1
154	10489629	Cdh22	6,28E-05	7,13E-04	2,1
155	10562399	Kctd15	1,13E-06	3,51E-05	2,1
156	10450242	C4b : C4a	8,96E-06	1,74E-04	2,1
157	10501903	Synpo2	1,27E-06	3,82E-05	2,1
158	10464775	Lrfn4	1,87E-08	1,48E-06	2,1
159	10516259	Dnali1	2,64E-06	6,73E-05	2,1
160	10578623	Wwc2	6,62E-06	1,37E-04	2,1
161	10387255	Hes7	2,24E-07	9,97E-06	2,1
162	10549276	Bhlhe41	3,09E-07	1,26E-05	2,0
163	10481491	Ier5l	1,23E-07	6,45E-06	2,0
164	10461869	Prune2	9,87E-06	1,86E-04	2,0
165	10389752	Nog	1,23E-04	0,001168822	2,0
166	10512807	Gabbr2	1,77E-05	2,87E-04	2,0
167	10401244	Actn1	4,73E-07	1,77E-05	2,0
168	10407766	Lgals8	1,27E-09	1,99E-07	2,0
169	10388430	Serpinf1	1,96E-05	3,09E-04	2,0
170	10356932	D1Ertd622e	2,19E-08	1,68E-06	2,0
171	10433618	Shisa9	5,55E-09	5,94E-07	2,0
172	10439483	Arhgap31	1,98E-07	9,16E-06	2,0
173	10357833	Atp2b4	1,65E-04	0,001444985	2,0
174	10510191	Zfp600	0,00750944	0,024912223	2,0
175	10513112	Epb4.1l4b	1,12E-07	5,94E-06	2,0
176	10536494	Cav2	1,67E-06	4,69E-05	2,0
177	10363231	Smpdl3a	1,45E-09	2,14E-07	2,0
178	10521678	Cd38	1,06E-07	5,77E-06	2,0
179	10553537	Luzp2	9,45E-04	0,0052941	2,0
180	10345550	Vwa3b	3,41E-05	4,55E-04	2,0
181	10407876	5033411D12Rik	7,17E-07	2,48E-05	2,0
182	10587880	Pcolce2	3,09E-05	4,25E-04	2,0
183	10462957	Tbc1d12	4,34E-07	1,65E-05	2,0
184	10561104	Axl	7,82E-07	2,64E-05	2,0
185	10598493	Pcsk1n	6,89E-08	4,08E-06	2,0
186	10592535	Sorl1	1,64E-07	7,99E-06	2,0
187	10395733	Npas3	5,26E-07	1,92E-05	2,0
188	10574018	Mt3	1,40E-10	4,37E-08	2,0
189	10441361	Tfb1m : Tiam2	2,12E-07	9,63E-06	2,0
190	10365482	Timp3	1,08E-04	0,001062427	2,0
191	10601874	Tceal3 : Tceal6 : Tc	2,15E-05	3,30E-04	2,0
192	10470014	Entpd2	9,01E-05	9,30E-04	2,0

193	10400304	Egln3	1,01E-04	0,001011072	2,0
194	10411107	Cmya5 : Gm4814	9,85E-07	3,14E-05	2,0
195	10407792	Gpr137b-ps : Gpr13	1,76E-08	1,42E-06	1,9
196	10498952	Gucy1a3	0,001304728	0,006741965	1,9
197	10427683	Spef2	1,99E-05	3,11E-04	1,9
198	10519105	Ski	8,55E-06	1,68E-04	1,9
199	10420935	Ephx2	2,58E-06	6,61E-05	1,9
200	10407803	Gpr137b	1,70E-07	8,23E-06	1,9
201	10514939	Podn	8,90E-07	2,90E-05	1,9
202	10602221	Mir680-2	1,18E-05	2,13E-04	1,9
203	10575160	Nfat5	1,37E-07	6,99E-06	1,9
204	10447602	Ezr	4,27E-07	1,63E-05	1,9
205	10475544	Sema6d	2,31E-08	1,73E-06	1,9
206	10511631	Slc26a7	8,49E-07	2,81E-05	1,9
207	10578222	A730069N07Rik : D	3,73E-06	8,81E-05	1,9
208	10566043	Lrrc51	2,57E-07	1,10E-05	1,9
209	10530870	Epha5	6,74E-06	1,39E-04	1,9
210	10397606	Tshr	3,45E-04	0,002490002	1,9
211	10433988	Serpind1	1,93E-07	9,02E-06	1,9
212	10494781	Igsf3	1,79E-06	4,97E-05	1,9
213	10438854	Atp13a4	2,22E-05	3,37E-04	1,9
214	10528183	Speer4d : Speer4c :	3,18E-06	7,75E-05	1,9
215	10419578	Ndrg2	1,32E-06	3,93E-05	1,9

List of the top 215 downregulated genes

			WT 12h fibrinogen vs. WT untreated		
Rank	ID-Ref	Gene Symbol	Fold-Change	P-value	Q-value
1	10487480	Bub1	13,6	1,59E-11	1,51E-08
2	10385248	Hmmr	13,4	7,94E-11	2,97E-08
3	10378053	Fam64a	13,2	5,04E-10	1,09E-07
4	10557156	Plk1	12,3	1,73E-09	2,49E-07
5	10462796	Kif11	11,3	1,93E-10	5,42E-08
6	10377405	Aurkb	11,0	1,29E-12	7,18E-09
7	10497831	Ccna2	10,4	3,27E-09	4,00E-07
8	10390707	Top2a	10,3	1,35E-09	2,04E-07
9	10454709	Kif20a : Cdc23	10,3	2,49E-11	1,69E-08
10	10601011	Kif4	10,0	5,74E-10	1,17E-07
11	10448506	Ccnf	9,8	9,57E-12	1,40E-08
12	10515836	Ccnb1	9,8	1,14E-08	1,03E-06
13	10400589	C79407	9,6	2,46E-11	1,69E-08
14	10420426	F630043A04R	9,5	9,22E-12	1,40E-08
15	10554445	Prc1	9,5	2,52E-09	3,33E-07
16	10563883	Depdc1a	9,3	3,90E-12	1,31E-08
17	10420877	Esco2	9,2	2,25E-11	1,69E-08
18	10515431	Kif2c	9,0	7,64E-11	2,97E-08
19	10497520	Ect2	8,7	6,90E-09	7,14E-07
20	10496204	Cenpe	8,7	1,14E-10	3,87E-08
21	10477187	Tpx2	8,5	1,34E-09	2,03E-07
22	10419323	Dlgap5	8,2	1,26E-10	4,07E-08
23	10462866	Cep55	8,2	8,53E-12	1,40E-08
24	10369815	Cdk1	7,9	1,19E-11	1,44E-08
25	10350392	Aspm	7,8	2,30E-10	5,95E-08
26	10474769	Bub1b	7,8	6,35E-10	1,28E-07
27	10371770	Gas2l3	7,8	2,80E-10	6,88E-08
28	10521731	Ncapg	7,8	4,61E-12	1,31E-08
29	10404063	Hist1h2ab	7,7	5,04E-11	2,31E-08
30	10474875	Casc5	7,7	1,34E-11	1,44E-08
31	10451805	Sgol1	7,6	9,87E-12	1,40E-08
32	10460738	Cdca5	7,6	5,17E-12	1,34E-08
33	10458195	Cdc25c	7,6	2,29E-10	5,95E-08
34	10350838	2810417H13R	7,5	1,06E-12	7,18E-09
35	10507112	Stil	7,4	1,47E-11	1,44E-08
36	10394978	Rrm2	7,4	1,65E-11	1,52E-08
37	10483401	Spc25	7,4	1,88E-11	1,62E-08
38	10568150	Kif22	7,3	1,45E-11	1,44E-08
39	10515744	Cdc20	7,2	1,72E-10	4,99E-08
40	10416037	Pbk	7,2	3,58E-09	4,30E-07
41	10578690	Neil3	7,1	2,87E-11	1,77E-08
42	10587508	Ttk	7,0	4,43E-11	2,17E-08

43	10432511	Racgap1	7,0	1,86E-09	2,64E-07
44	10594251	Kif23	6,9	7,67E-12	1,40E-08
45	10359890	Nuf2	6,9	3,41E-10	7,88E-08
46	10346365	Sgol2	6,8	2,03E-10	5,60E-08
47	10486255	Oip5 : Nusap1	6,7	2,55E-11	1,69E-08
48	10436106	C330027C09R	6,7	7,84E-11	2,97E-08
49	10490104	Aurka	6,6	1,52E-12	7,18E-09
50	10590494	Kif15	6,5	1,35E-11	1,44E-08
51	10414315	Cdkn3	6,5	8,38E-10	1,54E-07
52	10421029	Cdca2	6,5	3,83E-11	1,99E-08
53	10575733	Cenpn	6,3	1,20E-11	1,44E-08
54	10568714	Mki67	6,3	1,44E-08	1,22E-06
55	10520521	Cenpa	6,3	4,44E-10	9,71E-08
56	10389606	Prr11	6,3	6,68E-12	1,40E-08
57	10391811	Kif18b	6,2	2,36E-10	6,03E-08
58	10516246	Cdca8	6,2	1,47E-10	4,55E-08
59	10591781	Anln	6,2	4,18E-12	1,31E-08
60	10576883	Shcbp1	6,2	3,78E-10	8,59E-08
61	10405185	Cks2	6,1	7,95E-11	2,97E-08
62	10504470	Melk	5,8	1,37E-11	1,44E-08
63	10487340	Ncaph	5,8	3,87E-13	5,50E-09
64	10361375	Fbxo5	5,8	1,73E-09	2,49E-07
65	10360985	Cenpf	5,7	5,26E-10	1,12E-07
66	10528077	Dbf4	5,6	3,26E-11	1,89E-08
67	10541729	Cdca3	5,6	5,01E-09	5,42E-07
68	10426669	Troap	5,5	3,53E-11	1,97E-08
69	10371591	Pmch : 49305	5,5	8,39E-11	3,10E-08
70	10594774	Ccnb2	5,5	5,20E-07	1,90E-05
71	10563780	E2f8	5,5	2,85E-12	1,16E-08
72	10480432	Mastl	5,5	1,99E-10	5,56E-08
73	10485963	Arhgap11a	5,5	2,93E-11	1,77E-08
74	10352767	Nek2	5,4	5,29E-10	1,12E-07
75	10524169	Pole	5,4	3,77E-11	1,99E-08
76	10572906	Mcm5	5,4	3,22E-10	7,57E-08
77	10573261	Asf1b	5,4	2,45E-10	6,15E-08
78	10462632	Kif20b	5,3	1,82E-11	1,62E-08
79	10478572	Tnnc2 : Ube2c	5,2	6,08E-08	3,67E-06
80	10485979	Gjd2	5,2	2,11E-10	5,67E-08
81	10526952	Gpr30	5,1	2,46E-11	1,69E-08
82	10542079	4933413G19R	5,1	2,07E-10	5,67E-08
83	10487577	Ckap2l	5,1	1,17E-09	1,90E-07
84	10491805	Plk4	5,0	1,75E-10	5,03E-08
85	10399391	Gen1	5,0	2,98E-10	7,13E-08
86	10350297	Kif14	5,0	2,87E-09	3,68E-07
87	10538832	Mad2l1	4,9	6,12E-11	2,55E-08
88	10452709	Ndc80	4,9	1,58E-08	1,32E-06
89	10601705	Cenpi	4,8	1,72E-10	4,99E-08
90	10485982	Actc1	4,8	1,81E-06	5,01E-05
91	10450374	D17H6S56E-5	4,8	1,96E-08	1,53E-06
92	10474381	Kif18a	4,8	8,91E-10	1,59E-07

93	10461723	Fam111a	4,8	2,95E-09	3,75E-07
94	10474825	D2Ertd750e	4,8	1,13E-07	5,98E-06
95	10382998	Birc5	4,6	8,59E-12	1,40E-08
96	10540738	Fancd2	4,6	1,37E-10	4,32E-08
97	10465861	Incenp	4,5	5,92E-11	2,53E-08
98	10366983	Tmem194	4,5	7,47E-11	2,95E-08
99	10554325	5730590G19R	4,5	5,47E-11	2,39E-08
100	10426016	Gtse1	4,4	5,87E-10	1,19E-07
101	10525591	Kntc1	4,4	6,93E-10	1,38E-07
102	10351047	Cenpl	4,4	2,05E-11	1,68E-08
103	10594426	Zwilch : Lctl	4,3	7,84E-10	1,47E-07
104	10361995	Fam54a	4,3	2,13E-10	5,67E-08
105	10399087	Ncapg2	4,2	2,53E-09	3,33E-07
106	10584710	H2afx	4,2	4,55E-11	2,19E-08
107	10416736	6720463M24I	4,2	8,04E-10	1,48E-07
108	10406968	Cenpk : Ppwd	4,2	1,25E-11	1,44E-08
109	10504957	Smc2	4,2	4,35E-10	9,60E-08
110	10565570	4632434I11Ri	4,2	1,31E-10	4,20E-08
111	10363743	Rtkn2	4,2	2,38E-10	6,03E-08
112	10499639	Cks1b	4,1	8,68E-13	7,18E-09
113	10393662	Nptx1	4,1	1,32E-09	2,02E-07
114	10547943	Ncapd2	4,1	8,77E-10	1,59E-07
115	10592154	Hyls1	4,1	3,84E-11	1,99E-08
116	10450957	Cenpq	4,1	1,59E-10	4,81E-08
117	10604542	Hs6st2	4,0	2,64E-10	6,54E-08
118	10392374	Cacng5	4,0	2,10E-09	2,88E-07
119	10493137	Iqgap3	4,0	9,33E-11	3,36E-08
120	10577508	Ckap2	3,9	4,81E-07	1,79E-05
121	10467529	Opalin	3,9	2,14E-08	1,65E-06
122	10404061	Hist1h2bb	3,9	1,25E-09	1,98E-07
123	10423520	Sema5a	3,8	1,16E-06	3,58E-05
124	10467637	Arhgap19	3,7	1,16E-09	1,89E-07
125	10443007	Neurl1B	3,7	2,80E-11	1,77E-08
126	10428763	Atad2	3,7	3,17E-09	3,94E-07
127	10455813	Lmnb1	3,7	8,57E-11	3,12E-08
128	10592201	Chek1	3,7	2,38E-11	1,69E-08
129	10361110	Dtl	3,7	3,21E-11	1,89E-08
130	10384373	Fignl1	3,6	1,25E-07	6,49E-06
131	10351551	Adamts4	3,6	1,20E-08	1,07E-06
132	10474902	Rad51	3,6	1,28E-09	1,99E-07
133	10453715	Rab18	3,6	5,40E-10	1,12E-07
134	10446074	Uhrf1	3,6	3,33E-07	1,34E-05
135	10521090	Tacc3	3,5	6,69E-11	2,70E-08
136	10456904	Pstpip2	3,5	5,22E-05	6,27E-04
137	10350090	Ube2t	3,5	1,87E-09	2,65E-07
138	10373680	Neurod4	3,5	1,83E-07	8,65E-06
139	10497503	Kpna2	3,5	2,25E-11	1,69E-08
140	10594289	Glce	3,4	5,37E-10	1,12E-07
141	10430389	Mfng	3,4	6,73E-11	2,70E-08
142	10556266	Wee1	3,4	7,69E-09	7,71E-07

143	10368317	Enpp3	3,4	8,68E-09	8,48E-07
144	10511429	Car8	3,4	7,05E-09	7,27E-07
145	10546163	Mcm2	3,3	8,78E-10	1,59E-07
146	10430825	Cenpm	3,3	4,28E-08	2,78E-06
147	10377215	Gas7	3,3	3,38E-08	2,30E-06
148	10411728	Cenph	3,3	2,46E-10	6,15E-08
149	10464400	E330013P04R	3,2	1,07E-10	3,75E-08
150	10396402	Prkch	3,2	2,07E-08	1,60E-06
151	10524790	Cit	3,2	1,26E-10	4,07E-08
152	10427166	Espl1	3,1	7,23E-10	1,40E-07
153	10390050	Eme1	3,1	3,13E-10	7,42E-08
154	10571680	Ccdc111 : Mlf:	3,1	4,39E-09	4,97E-07
155	10571870	Hmgb2	3,1	3,85E-10	8,63E-08
156	10488382	Cd93	3,1	1,27E-05	2,24E-04
157	10408081	Hist1h1b	3,1	3,38E-08	2,30E-06
158	10474002	Chrm4	3,1	1,65E-10	4,95E-08
159	10499366	Pmf1	3,1	3,67E-09	4,35E-07
160	10524266	Chek2	3,1	1,95E-09	2,74E-07
161	10574427	Impdh2	3,0	4,11E-06	9,49E-05
162	10385096	Kcnip1	3,0	6,67E-09	6,95E-07
163	10437040	Chaf1b	3,0	2,88E-10	7,00E-08
164	10476989	Gins1	3,0	6,17E-09	6,50E-07
165	10566723	Lmo1	3,0	1,28E-08	1,12E-06
166	10593233	Htr3a	3,0	2,60E-07	1,11E-05
167	10554281	Polg : Fanci	3,0	7,16E-09	7,35E-07
168	10546294	Nup210	3,0	9,77E-08	5,38E-06
169	10599627	Hprt	3,0	2,64E-08	1,93E-06
170	10438690	Rfc4 : Eif4a2	3,0	4,80E-11	2,24E-08
171	10416340	Gfra2	3,0	2,69E-09	3,47E-07
172	10459755	Ska1	2,9	2,26E-08	1,70E-06
173	10444911	Mdc1	2,9	1,66E-08	1,36E-06
174	10528915	Tyms-ps : Tym	2,9	2,32E-07	1,02E-05
175	10352267	Lin9	2,9	2,25E-10	5,94E-08
176	10550102	Lig1	2,9	2,37E-07	1,04E-05
177	10512489	E130306D19R	2,9	7,15E-08	4,17E-06
178	10395612	G2e3	2,9	3,76E-11	1,99E-08
179	10447649	LOC10003909	2,9	1,63E-07	7,94E-06
180	10555695	Rrm1	2,8	1,01E-09	1,73E-07
181	10586416	Pif1	2,8	3,93E-09	4,58E-07
182	10367076	Prim1	2,8	1,06E-09	1,77E-07
183	10509410	Rap1gap	2,8	7,32E-06	1,48E-04
184	10495574	Ccdc76 : Sass(	2,8	1,14E-10	3,87E-08
185	10455967	2610318N02R	2,8	4,91E-09	5,35E-07
186	10518927	Kcnab2	2,8	6,60E-09	6,90E-07
187	10555510	Pde2a	2,8	2,29E-05	3,45E-04
188	10406581	Msh3 : Dhfr	2,8	1,67E-08	1,36E-06
189	10451225	Polh	2,8	4,18E-08	2,73E-06
190	10564978	Blm	2,8	2,25E-08	1,70E-06
191	10403943	Hist1h2bm	2,8	8,84E-10	1,59E-07
192	10508151	Clspn	2,7	9,46E-08	5,26E-06

193	10409424	Mxd3	2,7	3,98E-07	1,54E-05
194	10523923	Ccdc18	2,7	7,53E-08	4,35E-06
195	10548086	Rad51ap1	2,7	2,42E-06	6,33E-05
196	10418895	Zfp488	2,7	6,46E-05	7,27E-04
197	10523758	Lrrc8b	2,7	7,11E-10	1,39E-07
198	10466779	Pip5k1b	2,7	3,16E-04	0,00232452
199	10582809	Tk1	2,7	1,31E-07	6,76E-06
200	10381096	Igfbp4	2,7	3,09E-07	1,26E-05
201	10473125	Itga4	2,7	1,70E-08	1,38E-06
202	10469936	Nrarp	2,7	5,36E-06	1,17E-04
203	10485213	Cd82	2,7	6,06E-06	1,28E-04
204	10479811	Mcm10	2,7	7,74E-09	7,72E-07
205	10482500	Rnd3	2,7	9,39E-08	5,25E-06
206	10423109	Adamts12	2,7	3,15E-09	3,94E-07
207	10479698	Myt1	2,7	1,95E-04	0,001624
208	10490129	Bmp7	2,6	3,47E-09	4,21E-07
209	10428222	Ncald	2,6	7,98E-09	7,88E-07
210	10557519	Hirip3	2,6	1,40E-07	7,10E-06
211	10604837	Sox3	2,6	1,68E-08	1,36E-06
212	10498620	Trim59	2,6	2,48E-07	1,07E-05
213	10448615	Rab26 : Traf7	2,6	5,35E-08	3,30E-06
214	10360745	Lbr	2,6	1,33E-09	2,02E-07
215	10430748	Rangap1	2,6	1,01E-09	1,73E-07
