# Supplementary material for: Fibrinogen induces neural stem cell differentiation into astrocytes in the subventricular zone via BMP signaling
Source: Nat Commun. 2020 Jan 31;11:630. doi: 10.1038/s41467-020-14466-y (PMC6994610; doi:10.1038/s41467-020-14466-y)
Supplement: Supplementary file 3 — Reporting Summary [file 41467_2020_14466_MOESM3_ESM.pdf]

nature research

Corresponding author(s): Schachtrup, Christian

Last updated by author(s): Dec 16, 2019

Reporting Summary

Nature Research wishes to improve the reproducibility of the work that we publish. This form provides structure for consistency and transparency in reporting. For further information on Nature Research policies, see Authors & Referees and the Editorial Policy Checklist.

Statistics

For all statistical analyses, confirm that the following items are present in the figure legend, table legend, main text, or Methods section.

n/a	Confirmed	
☐	☒	The exact sample size (n) for each experimental group/condition, given as a discrete number and unit of measurement
☐	☒	A statement on whether measurements were taken from distinct samples or whether the same sample was measured repeatedly
☐	☒	The statistical test(s) used AND whether they are one- or two-sided<br>*Only common tests should be described solely by name; describe more complex techniques in the Methods section.*
☒	☐	A description of all covariates tested
☒	☐	A description of any assumptions or corrections, such as tests of normality and adjustment for multiple comparisons
☐	☒	A full description of the statistical parameters including central tendency (e.g. means) or other basic estimates (e.g. regression coefficient) AND variation (e.g. standard deviation) or associated estimates of uncertainty (e.g. confidence intervals)
☒	☐	For null hypothesis testing, the test statistic (e.g. F, t, r) with confidence intervals, effect sizes, degrees of freedom and P value noted<br>*Give P values as exact values whenever suitable.*
☒	☐	For Bayesian analysis, information on the choice of priors and Markov chain Monte Carlo settings
☒	☐	For hierarchical and complex designs, identification of the appropriate level for tests and full reporting of outcomes
☒	☐	Estimates of effect sizes (e.g. Cohen's d, Pearson's r), indicating how they were calculated

Our web collection on statistics for biologists contains articles on many of the points above.

Software and code

Policy information about availability of computer code

Data collection	GraphPad Prism 7, AxioVision image analysis software (Carl Zeiss), ImageJ (http://rsbweb.nih.gov/ij/), Q-PCR was performed using Applied Biosystems analysis software
Data analysis	Statistics were performed by using GraphPad Prism software (GraphPad Software Inc., San Diego, USA).

For manuscripts utilizing custom algorithms or software that are central to the research but not yet described in published literature, software must be made available to editors/reviewers. We strongly encourage code deposition in a community repository (e.g. GitHub). See the Nature Research guidelines for submitting code & software for further information.

Data

Policy information about availability of data

All manuscripts must include a data availability statement. This statement should provide the following information, where applicable:

- Accession codes, unique identifiers, or web links for publicly available datasets
- A list of figures that have associated raw data
- A description of any restrictions on data availability

Gene array data reported in this paper are available at NCBI Gene expression Omnibus with accession numbers E-MTAB-3013. All other data are available upon reasonable request.

Field-specific reporting

Please select the one below that is the best fit for your research. If you are not sure, read the appropriate sections before making your selection.

☒ Life sciences ☐ Behavioural & social sciences ☐ Ecological, evolutionary & environmental sciences

For a reference copy of the document with all sections, see nature.com/documents/nr-reporting-summary-flat.pdf

Life sciences study design

All studies must disclose on these points even when the disclosure is negative.

Sample size

Sample size calculations are based on previously published data as stated for the different experiments in the Methods or Results sections. No statistical methods were used to predetermine sample sizes.

Data exclusions

No data points were excluded.

Replication

All number of replication of the experiments are detailed in the figure legends. Sample size calculations are based in previously published data as stated for the different experiments in the Methods or results sections

Randomization

For pharmacologic treatments in vivo, mice were randomly divided into 2 groups, and one group received the pharmacologic reagent and the other group the control solution, as it is stated in the method section. Cells were all plated at the same time and wells randomly selected for treatments with growth factors or inhibitors.

Blinding

Investigators were not blinded to group allocation during data collection.

Reporting for specific materials, systems and methods

We require information from authors about some types of materials, experimental systems and methods used in many studies. Here, indicate whether each material, system or method listed is relevant to your study. If you are not sure if a list item applies to your research, read the appropriate section before selecting a response.

Materials & experimental systems

n/a	Involved in the study
☐	☒ Antibodies
☒	☐ Eukaryotic cell lines
☒	☐ Palaeontology
☐	☒ Animals and other organisms
☒	☐ Human research participants
☒	☐ Clinical data

Methods

n/a	Involved in the study
☒	☐ ChIP-seq
☒	☐ Flow cytometry
☒	☐ MRI-based neuroimaging

Antibodies

Antibodies used

Antibodies used were as follows: AQP4 (H-80) (SCBT, sc-20812), anti-BrdU (Abcam ab6326), CD31 (Abcam ab28364), CD13 (R&D Systems, AF2335), CSPG (Sigma, C8035), Doublecortin (Millipore AB2253), δ-GFAP (Millipore, AB9598), GFAP (Abcam, ab7260), GFAP (Invitrogen, 13-0300), GFP (Abcam, ab5450), rabbit anti-GFP (Abcam, ab290), Fibrinogen (USBiological, F4203-02C), Fibrinogen (USBiological, F4203-02F), rabbit anti-Id3 (Calbioreagent, M101), Lcn2 (R&D Systems, AF1857-SP), Nestin (SBTC, sc-21249), Nestin (Antibodies-Online, ABIN188165), NeuN (Abcam, ab177487), S100β (Abcam, ab52642), Thbs4 (Genetex, GTX87694), Thbs4 (R&D Systems, AF7860), Aldhl1l (Abcam, ab87117), β1-integrin (9EG7, BD Bioscience, 553715) P-smad1/5/8 (Cell Signaling, 13820), Tuj-1 (Abcam, ab18207), Tuj-1 (Millipore, MAB1637), aldolase C (SCBT, sc-271593), Smad1 (Cell Signaling, 9743), GAPDH (Cell Signaling, 2118).

Validation

Literature is cited in the Methods section or can be found on the companies website.

Animals and other organisms

Policy information about studies involving animals; ARRIVE guidelines recommended for reporting animal research

Laboratory animals

For animal experiments, C57BL/6 mice (Charles River) and C57BL/6J-inbred mice deficient for Fgα-/- were used. For analysis of SVZ NSPCs, Nestin-CreERT2 mice40 were crossed with YFPfl mice, resulting in Nestin-CreERT2;YFPfl mice. C57BL/6J-inbred mice deficient for inhibitor of DNA binding 3 (Id3−/−) were used for NSPC isolation and culture. Animals of both sex have been used and 8-12 week old aged matched mice were used.

Wild animals

No wild animal was used for this study.

Field-collected samples	This study did not involve sample collected from the field.
Ethics oversight	All animal experiments were approved by the Federal Ministry for Nature, Environment and Consumers Protection of the state of Baden-Württemberg and were performed in accordance to the respective national, federal and institutional regulations.

Note that full information on the approval of the study protocol must also be provided in the manuscript.
